# Moxibustion for Diarrhea-Predominant Irritable Bowel Syndrome: A Systematic Review and Meta-Analysis of Randomized Controlled Trials

**DOI:** 10.1155/2016/5105108

**Published:** 2016-05-16

**Authors:** Bozong Tang, Jianliang Zhang, Zongguo Yang, Yunfei Lu, Qingnian Xu, Xiaorong Chen, Jiang Lin

**Affiliations:** ^1^Shuguang Hospital, Shanghai University of Traditional Chinese Medicine, Shanghai 200021, China; ^2^Shanghai Public Health Clinical Center, Shanghai 201508, China

## Abstract

*Background*. The complementary and alternative medicines in treatment of diarrhea-predominant irritable bowel syndrome (IBS-D) are controversial.* Methods*. We searched PubMed, Ovid Embase, Web of Science, Cochrane Library databases, CNKI, Wanfang Database, CBM, VIP, and AMED for randomized controlled trials (RCTs) of moxibustion compared with pharmacological medications in patients with IBS-D. A meta-analysis was performed using both fixed and random-effects models based on heterogeneity across studies.* Results*. In total, 568 patients in 7 randomized controlled trials were randomly treated with moxibustion and pharmacological medications. The improvement of global IBS-D symptoms and overall scores was significant (*P* = 0.0001 and *P* < 0.0001, resp.) in patients treated by moxibustion only compared to pharmacological medications. The specific IBS-D symptoms of abdominal pain, abdominal distension, abnormal stool, and defecation frequency were alleviated in patients treated by moxibustion compared to pharmacological medications, but no significance was found except for abdominal distension and defecation frequency (*P* = 0.03 and *P* = 0.02, resp.). There were no serious adverse events related to moxibustion.* Conclusions*. Moxibustion appears to be effective in treating IBS-D compared with pharmacological medications. However, further large, rigorously designed trials are warranted due to insufficient methodological rigor in the included trials.

## 1. Introduction

Irritable bowel syndrome (IBS) is a chronic, recurrent functional gastrointestinal (GI) disorder characterized by altered bowel habits and abdominal pain or discomfort [1]. The prevalence of IBS ranges from 5% to 20% in the general population worldwide [2]. The high prevalence of IBS results in a substantial socioeconomic burden through decreased work productivity and quality of life and increased direct and indirect healthcare costs [3–5]. The direct and indirect healthcare costs related to IBS in the United States have been steadily increasing and amounted to 1.35 billion dollars in 2003 [6]. The worldwide health costs associated with IBS are estimated to exceed 200 billion dollars [7].

The etiology and pathophysiology of IBS remain less understood. Abnormal intestinal motility, visceral hypersensitivity, abnormal neurohormonal responses to stimuli or stress, and alteration of normal intestinal microflora are related to IBS [1]. The available western medications mainly target symptom relief, such as antispasmodics, fiber supplementation, and antidepressants. Due to limited therapeutic efficacy and the side effects of western medications, up to 51% of IBS patients, especially IBS-D patients, are interested in complementary and alternative medicine (CAM) [8, 9]. Moxibustion is a type of CAM approach that stimulates specific points to improve general health and treat chronic conditions with heat generated by burning dried mugwort (*Artemisia vulgaris*) leaves [10].

The average incidence of diarrhea-predominant IBS (IBS-D) is quite high and is showing an increasing trend; it seriously impacts the life quality of patients [11]. In CAM practice, most IBS-D patients have a deficiency of both the spleen and stomach, insufficiency of the kidney yang, and incoordination between the liver and the spleen which are suitable for moxibustion therapy [12]. Although a meta-analysis showed that moxibustion can improve global symptoms of IBS, no systematic study has evaluated the effectiveness of moxibustion treatment for IBS-D [13]. Moreover, some studies including acupuncture or pharmacological medications may influence results [14, 15].

Therefore, we conducted a systematic review and meta-analysis to evaluate all the currently available randomized controlled trials (RCTs) of moxibustion compared with pharmacological medications for symptom improvement in IBS-D patients.

## 2. Materials and Methods

### 2.1. Search Strategy

We searched the following electronic databases through March 2015: PubMed, Ovid Embase, Web of Science, and Cochrane Library databases, Chinese National Knowledge Infrastructure (CNKI) Database, Wanfang Database, Chinese Biomedical (CBM) Database, Chinese Science and Technology Periodical Database (VIP), and Allied and Complementary Medicine Database (AMED). We used a combination of medical subject headings without language limitation:* irritable bowel syndrome* (IBS),* diarrhea*,* diarrhea-predominant irritable bowel syndrome*,* moxibustion*,* moxibustion therapy*,* moxa-moxibustion*,* warm-moxibustion*,* complementary therapies*,* Chinese medicine*,* traditional medicine*,* alternative medicine*,* complementary medicine*,* randomized controlled trial*, and* controlled clinical trial*. Reference lists from trials selected by electronic searching and conference compilations were manually searched. The literature search was conducted by Bozong Tang and Zongguo Yang independently.

### 2.2. Study Selection

Two authors independently selected trials and discussed inconsistencies. Articles that met the following criteria were included: (1) randomized controlled trials; (2) patients with chronic IBS-D; (3) intervention that was moxibustion compared with western medications; (4) studies that measured improvement of symptoms or scores; and (5) available full text. Studies that included other treatments influencing the curative effect of moxibustion, including acupuncture and electroacupuncture, were excluded.

### 2.3. Data Extraction and Quality Assessment

Two reviewers screened all the retrieved trials independently and extracted the following content: publication data, study design, sample size, subject characteristics, treatment protocol, and outcome measurement. The methodological qualities of all the eligible RCTs were assessed independently by two reviewers according to Cochrane Collaboration's tool described in Handbook version 5.1.0 [22]. Two authors (Bozong Tang and Zongguo Yang) assessed the quality independently, and inconsistency was discussed with a third review author (Jiang Lin) who acted as an arbiter.

### 2.4. Statistical Methods

Data were processed in accordance with the Cochrane Handbook [22]. Intervention effects were presented with odds ratios (ORs) and 95% confidence intervals (CIs) for dichotomous data and mean differences (MDs) and 95% CIs for continuous data. Continuous data of subgroups of each study were pooled using the following formula [23]:

(1) $$\begin{array}{c} SD=\sqrt{\frac{\left(N_{1}-1\right){SD}_{1}^{2}+\left(N_{2}-1\right){SD}_{2}^{2}+\left(N_{1}N_{2}/\left(N_{1}+N_{2}\right)\right)\left(M_{1}^{2}+M_{2}^{2}-2M_{1}M_{2}\right)}{N_{1}+N_{2}-1}}, \end{array}$$

where SDs were the standard deviations, *N*s were the sample sizes, and *M*s were the means.

Heterogeneity across studies was informally assessed by visual inspection of forest plots and formally estimated by Cochran's *Q* test, which uses a chi-square distribution to make inferences about the null hypothesis of homogeneity (considered significant at *P* < 0.10). A rough guide to our interpretation of *I* ^2^ was as follows:

- At 0–40%, it may not be important.
- At 30–60%, it may represent moderate heterogeneity.
- At 50–90%, it may represent substantial heterogeneity.
- At 75–100%, it reflects considerable heterogeneity [22, 24].

If the eligibility of any study in the meta-analysis was dubious because of incomplete data, a sensitivity analysis was performed. If there was no heterogeneity among the trials, a fixed effects model would be applied in a meta-analysis. If there was heterogeneity among the trials, a random-effects model would be used instead in the meta-analysis. A description analysis was performed if the quantitative data could not be pooled. Review Manage (RevMan) version 5.3 software was used for data analysis.

## 3. Results

### 3.1. Study and Patient Characteristics

**Figure 1:**
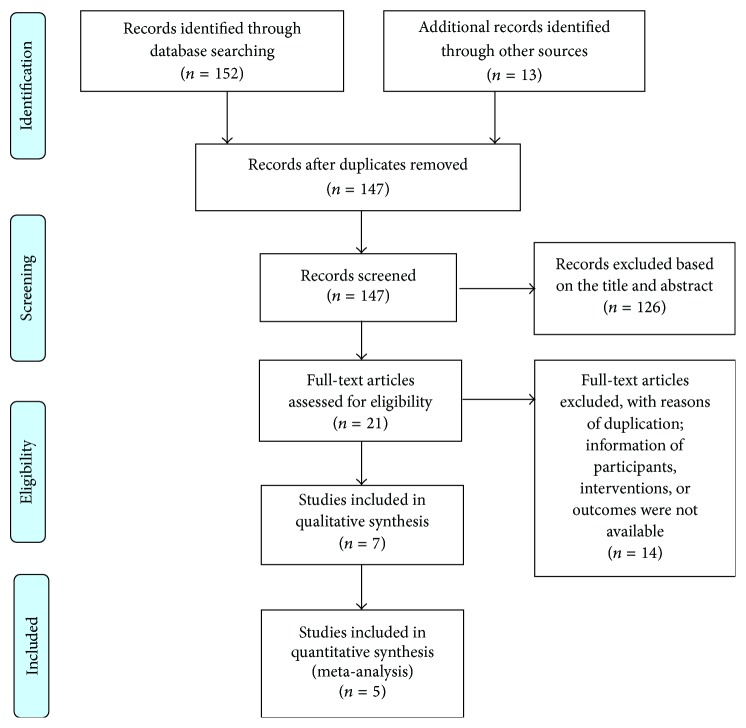
Flow diagram of the study selection process.

After primarily searching in 7 databases, 165 papers were found. However, 144 papers were excluded due to ineligibility after reviewing the titles and the abstracts. Additional 14 papers were excluded due to duplication and unavailable information on participants, interventions, and outcomes. Finally, 7 randomized controlled trials [12, 16–21] were included in this review: 3 trials published in English journals and 4 trials published in Chinese journals (Figure 1). A total of 568 patients were randomly treated with moxibustion or a pharmacological medication. The baseline characteristics of patients included in this meta-analysis are described in Table 1.

### 3.2. Methodological Quality Assessment

**Figure 2:**
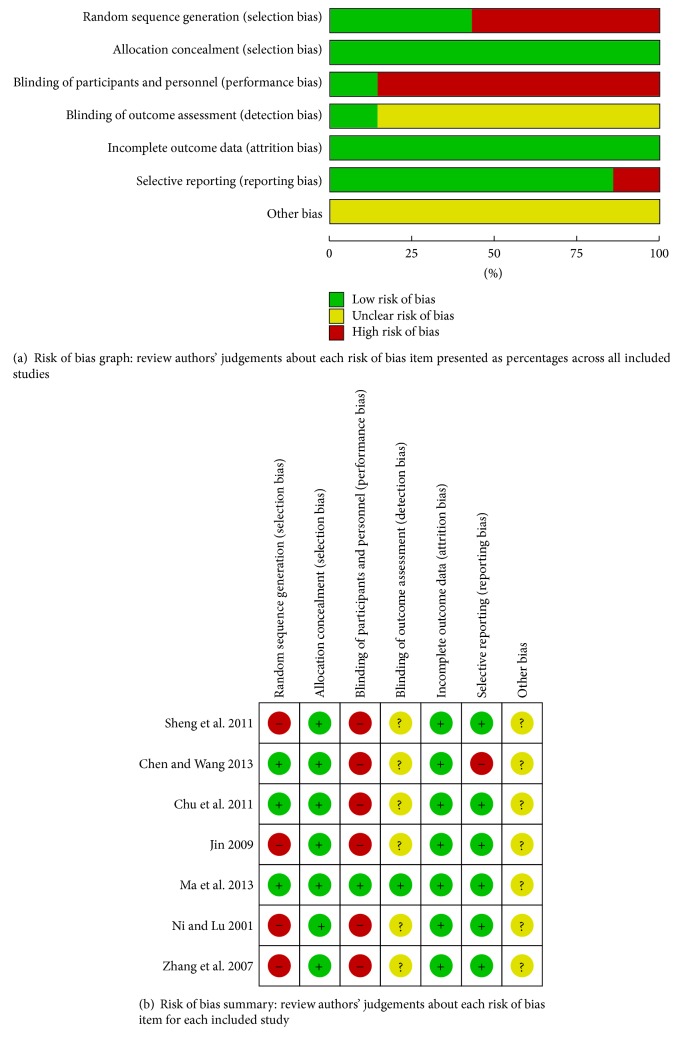
Risk of bias assessment.

All studies included in this meta-analysis were randomized controlled trials. Four studies [12, 16–18] did not report the method of randomization, whereas the other three reported a randomization number sequence or adaptive minimization randomization scheme [19–21]. Except for Ma et al.'s study using the single blind method, all the other studies did not adopt a blind method. These studies had high performance bias and detection bias. Selective reporting was found in Chen and Wang's study [20] because it did not present the ITT analysis data. The other potential biases were unclear in these trials (Figure 2). Because all the studies were conducted in China and clinical outcomes of overall IBS-D symptoms and scores were subjective, we cautiously drew the conclusion that publication bias might have been present in this meta-analysis.

### 3.3. Overall IBS-D Symptoms or Scores

**Figure 3:**
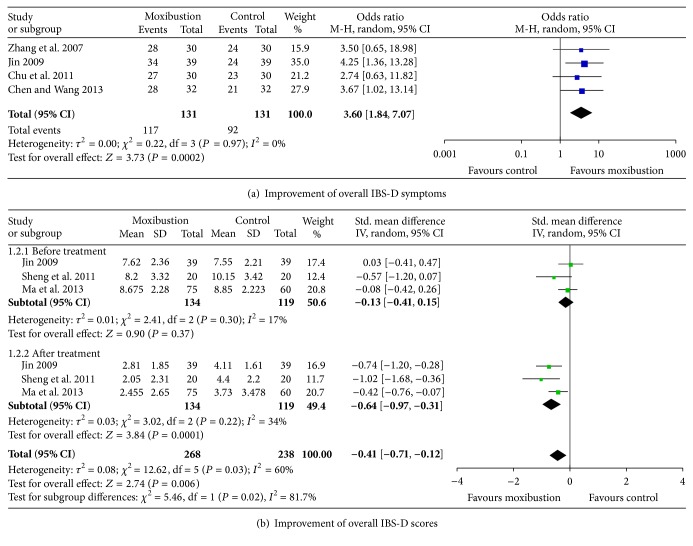
Improvement of overall IBS-D symptoms and scores.

The efficacy of moxibustion treatment alone was compared with that of pharmacological medication treatment in 7 studies [12, 16–21]. Improvement of global IBS-D symptoms was reported in 4 studies [16, 17, 19, 20], and improvement of IBS-D scores was reported in the other 3 studies [17, 18, 21]. There was no significant heterogeneity among the included studies [16–21] (*P* = 0.97, *I* ^2^ = 0%). A random-effect model was applied to compare the efficacy of moxibustion treatment and medication treatment. The effects of moxibustion on the improvement of the effective rate of overall IBS-D symptoms and the overall IBS-D symptoms scores were both superior to those of medication (*P* = 0.0002, Figure 3(a), and *P* = 0.0001, Figure 3(b)).

### 3.4. Specific IBS-D Symptoms

**Figure 4:**
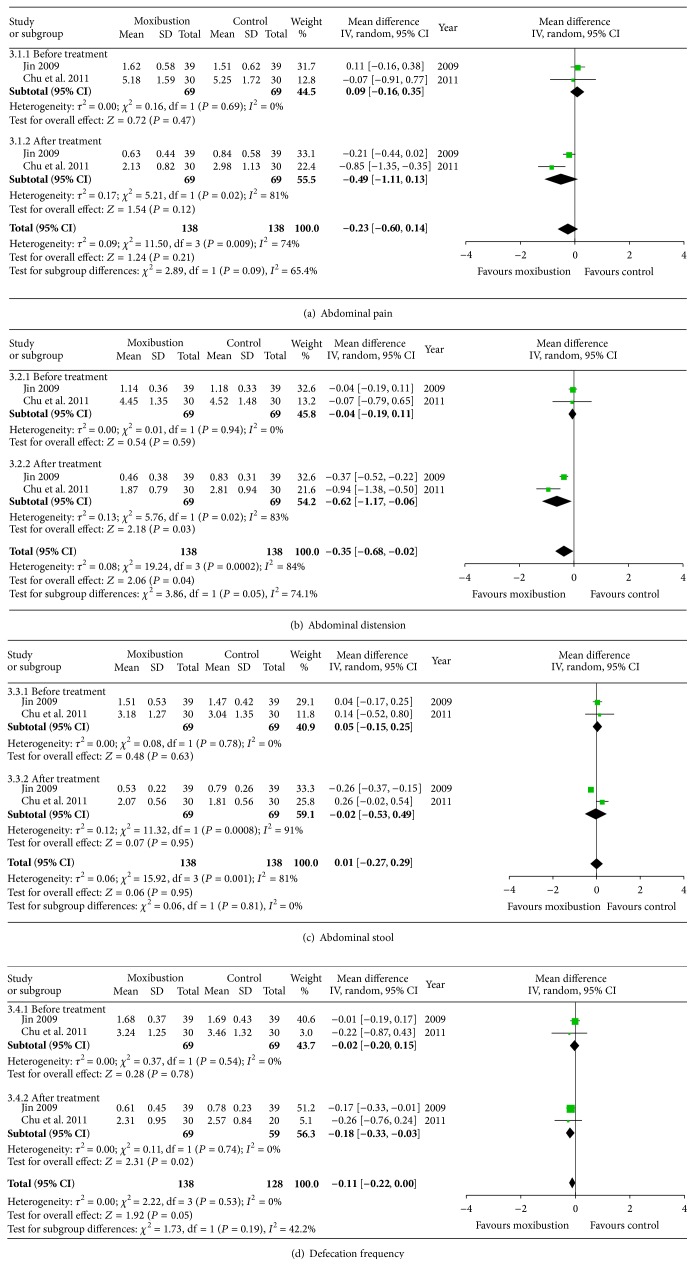
Improvement of specific IBS-D symptoms.

Improvement of specific IBS-D symptoms such as abdominal pain, abdominal distension, abnormal stool, and defecation frequency was reported in 2 studies [17, 19]. The heterogeneity of abdominal pain, abdominal distension, abnormal stool, and defecation frequency among the included studies was not significant before treatment (*P* = 0.69, *P* = 0.94, *P* = 0.78, and *P* = 0.54, resp.). However, the heterogeneity of the specific symptoms, except for defecation frequency, was significant after treatment. Thus, a random-effects model was applied to compare the efficacy of moxibustion treatment and medication treatment. There was no significant difference in improvement of abdominal pain and abnormal stool between the two treatments (*P* = 0.21 and *P* = 0.95, Figures 4(a) and 4(c)). However, the improvement of abdominal distension and defecation frequency with moxibustion treatment was superior to medication treatment (*P* = 0.02 and *P* = 0.02, Figures 4(b) and 4(d)).

### 3.5. Adverse Events

Only one trial reported two cases of mild-to-moderate allergy related to moxibustion, which disappeared after stopping the treatment [21]. The other six trials did not report adverse events.

## 4. Discussion

IBS is a functional gastrointestinal disorder characterized by chronic or recurrent abdominal pain and/or abdominal discomfort associated with abnormal bowel movement [1]. The diagnosis of IBS is currently based on the presence of characteristic symptoms (abdominal pain/discomfort, bloating/distension, and alterations in defecatory function) and in the absence of organic diseases of the gastrointestinal tract [25, 26]. According to the symptoms, IBS can be divided into different subtypes. Based on the Rome III diagnostic criteria that is currently widely used, IBS is classified into four subtypes including IBS-D, IBS-C (constipation-predominant), IBS-M (mixed), and IBS-U (unspecified), whereas IBS-D is the most common subtype in China [1, 27, 28].

The pathophysiology of IBS includes abnormal intestinal motility, visceral hypersensitivity, psychosocial distress, neuromodulation disorder in postinfection, and imbalanced gut microbiota [29]. Antispasmodics, antidiarrheals, 5-hydroxytryptamine 3 (5-HT_3_) receptor antagonist [30], probiotics [31], selective serotonin reuptake inhibitors [29], and antibiotics [32] are used to treat IBS-D. Antispasmodics plus stool consistency modifiers are the first-line options to treat the major symptoms and defecation. However, several systematic reviews conducted by the American College of Gastroenterology Task Force showed poor quality of evidence that particular antispasmodics and antidiarrheals can reduce defecation frequency but that they cannot affect the overall symptoms of IBS; 5-HT_3_ agonists carry a possible risk of ischemic colitis and cardiovascular events [1]. A meta-analysis reported that the response rate to placebo was 42.6%, which was similar to that of conventional pharmacological medication [33]. Therefore, the unsatisfactory therapeutic efficacy and side effects of conventional pharmacological medication are influencing researchers to try to find more effective and safer therapies in CAM.

Moxibustion is not only a treatment approach of CAM but also an important component of traditional Chinese medicine (TCM). There are several types of moxibustion including scarring moxibustion (burning moxa on the skin), warming moxibustion (burning moxa above the skin), and herb-partition moxibustion (indirect burning interposed by various materials). Warming moxibustion is the most practical and convenient approach in clinical practice [34]. According to TCM theory, moxibustion warms the interior and dissipates the cold, regulates qi and resolves stasis, softens and dissolves mass, resuscitates yang, and warms and activates the meridians. Previous studies indicate that moxibustion could relieve chronic visceral hyperalgesia (CVH) by activating the spinal dynorphin and orphanin-FQ system [34], decreasing hypothalamic corticotrophin releasing hormone levels [35], and decreasing prokineticin-1 and prokineticin receptor-1 expression [36]. Moxibustion also could enhance the pain threshold and restore sensitivity by decreasing 5-hydroxytryptamine concentration in the colon tissue [37]. A clinical study observed the change in colonic mucosal 5-HT_3_ among IBS-D patients and assessed the efficacy of herb-partitioned moxibustion. The results showed that IBS-D patients had a significantly increased expression of 5-HT_3_ in the colonic mucosa, whereas herb-partitioned moxibustion simultaneously improved IBS-D symptoms and downregulated the level of 5-HT_3_ [37].

Our meta-analysis showed that moxibustion could improve global IBS-D patient symptoms and scores, which was consistent with previous studies [38, 39]. In our meta-analysis, Jin and Chu et al. [17, 19] reported that moxibustion could relieve diarrhea and abdominal pain in IBS-D patients, which was in accordance with the results of Liu and Wu et al. [37, 40]. However, the improvement of abdominal pain and abnormal stool was not significantly different in our meta-analysis with moxibustion treatment, whereas abdominal distension and defecation frequency improved significantly. These findings might be associated with different frequencies of intervention, duration of study, patient age, duration of run-in period, male-to-female ratio, the number of patients in the treatment group or control group, or the number of doctor visits.

Systematic reviews and meta-analyses are often limited by the quality of the included studies. First, the sample size is small, in which only 568 patients were included in both moxibustion and pharmacological medication groups. Second, the treatment mode and the duration were not equivalent; thus, we could not confirm how long moxibustion treatment is required to achieve a benefit when treating IBS-D. Third, because the assessment of improved symptoms of IBS-D was not the same, it was difficult to accurately assess the effect of moxibustion. Fourth, because only one study reported the side effects of moxibustion, we could not assess the overall side effects during treatment of IBS-D. Fifth, the quality of the present evidence is limited considering that most of the included studies were given a high risk of performance bias for key methodological elements of adequate random sequence generation and allocation concealment. Finally, no studies reported an improvement in quality of life for IBS-D patients, which is correlated with the appearance of symptoms, protracted time, and severity of the disease [41, 42].

This meta-analysis showed that moxibustion might be beneficial for IBS-D patients. However, this review had some limitations. The data are insufficient to recommend the method as a first-line treatment or to establish the quality of life and long-term results. Therefore, further research is required to more accurately assess the results of moxibustion for treating IBS-D.

## Figures and Tables

**Table 1:** Baseline characteristics of included randomized controlled trials for IBS-D.

Study year	Number of patients	Publishing language	Diagnostic criteria	Criteria for improvement in overall IBS-D symptoms	Time point for outcome assessment	Moxibustion treatment(s)	Control treatment(s)
Ni and Lu 2001 [12]	56	English	Negative GI investigations and standards for clinical diagnosis for IBS from 1986 National conference for chronic diarrhea	Change of total IBS symptom score (predefined)	15 days (EoT)	Fixed points	Nifedipinum, 10 mg t.i.d.

Zhang et al. 2007 [16]	60	Chinese	Rome II	≥30% improvement in global IBS symptoms	2 weeks (EoT)	Ginger-partitioned and fixed points	Diet, psychiatric, and antidiarrheal therapy Enterosoluble glutamine 0.4 g t.i.d. or Smecta 3 g t.i.d. or probiotics 630 mg t.i.d.

Jin 2009 [17]	78	Chinese	Rome II	≥30% improvement in global IBS symptoms	30 days (EoT)	Traditional Chinese ointments-partitioned and fixed points	Berberine hydrochloride 2 tablets t.i.d.

Sheng et al. 2011 [18]	40	English	Rome III	≥30% improvement in global IBS symptoms	4 weeks (EoT and 1-month follow-up)	Herbal cone-partitioned and fixed points	Pinaverium bromide 50 mg t.i.d.

Chu et al. 2011 [19]	60	Chinese	Rome II and TCM criteria	≥30% improvement in global IBS symptoms	15 days (EoT)	Syndrome differentiation and treatment	Loperamide 2 mg b.i.d.

Chen and Wang 2013 [20]	64	Chinese	Rome III	≥50% improvement in global IBS symptoms	30 days (EoT)	Fixed points	Trimebutine maleate tablets 100 mg t.i.d.

Ma et al. 2013 [21]	210	English	Rome III	GSRS total score	4 weeks (EoT)	Medicamental pulverata-partitioned and fixed points	Pinaverium bromide 50 mg t.i.d.

IBS-D: diarrhea-predominant IBS; t.i.d.: three times a day; GSRS: gastrointestinal symptom rating scale; EoT: end of treatment; TCM: traditional Chinese medicine.

## References

[1] Brandt L. J., Chey W. D., Foxx-Orenstein A. E. (2009). An evidence-based position statement on the management of irritable bowel syndrome. *American Journal of Gastroenterology*.

[2] Rey E., Talley N. J. (2009). Irritable bowel syndrome: novel views on the epidemiology and potential risk factors. *Digestive and Liver Disease*.

[3] Hungin A. P. S., Chang L., Locke G. R., Dennis E. H., Barghout V. (2005). Irritable bowel syndrome in the United States: prevalence, symptom patterns and impact. *Alimentary Pharmacology & Therapeutics*.

[4] Drossman D. A., Morris C. B., Schneck S. (2009). International survey of patients with IBS: symptom features and their severity, health status, treatments, and risk taking to achieve clinical benefit. *Journal of Clinical Gastroenterology*.

[5] Ringel Y., Williams R. E., Kalilani L., Cook S. F. (2009). Prevalence, characteristics, and impact of bloating symptoms in patients with irritable bowel syndrome. *Clinical Gastroenterology and Hepatology*.

[6] Inadomi J. M., Fennerty M. B., Bjorkman D. (2003). Systematic review: the economic impact of irritable bowel syndrome. *Alimentary Pharmacology Therapeutics*.

[7] McFarland L. V. (2008). State-of-the-art of irritable bowel syndrome and inflammatory bowel disease research in 2008. *World Journal of Gastroenterology*.

[8] Carmona-Sánchez R., Tostado-Fernández F. A. (2005). Prevalence of use of alternative and complementary medicine in patients with irritable bowel syndrome, functional dyspepsia and gastroesophageal reflux disease. *Revista de gastroenterología de México*.

[9] Magge S., Lembo A. (2011). Complementary and alternative medicine for the irritable bowel syndrome. *Gastroenterology Clinics of North America*.

[10] Yoon S. L., Grundmann O., Koepp L., Farrell L. (2011). Management of irritable bowel syndrome (IBS) in adults: conventional and complementary/alternative approaches. *Alternative Medicine Review*.

[11] Hu D., Kang M., Xiong J., Deng P. (2012). Irritable bowel syndrome with diarrhea (IBS-D) treated with moxibustion on heat-sensitive acupoints: a randomized controlled trial. *World Journal of Acupuncture—Moxibustion*.

[12] Ni Y. Y., Lu J. Q. (2001). Clinical research on moxibustion treatment of diarrhea type irritable bowel syndrome. *World Journal of Acupuncture-Moxibustion*.

[13] Park J.-W., Lee B.-H., Lee H. (2013). Moxibustion in the management of irritable bowel syndrome: systematic review and meta-analysis. *BMC Complementary and Alternative Medicine*.

[14] Hu D., Kang M., Xiong J. (2012). Irritable bowel syndrome with diarrhea (IBS-D) treated with moxibustion on heat-sensitive acupoints: a randomized controlled trial. *World Journal of Acupuncture-Moxibustion*.

[15] Luo S. J., Long J. H., Huang L. (2008). The curative effect observation and nursing of moxibustion combined with pinaverium bromide tablets to cure abdominal pain and diarrhea intestine irritable syndrome. *Journal of Jinggangshan Medical College*.

[16] Zhang Y. B., Yan C. Y., Xie S. (2007). Thirty cases of diarrhea-predominant irritable bowel syndrome treated by ginger-partitioned moxibustion. *Jiangxi Journal of Traditional Chinese Medicine*.

[17] Jin G. D. (2009). The efficiency observation of tianjiu therapy on irritable bowel syndrome of diarrhea type. *Journal of Zhejiang College of Traditional Chinese Medicine*.

[18] Sheng C., Dongqing D., Ma Y., Wang Z., Gao S., Wang X. (2011). clinical study on herbal cone-partitioned moxibustion for irritable bowel syndrome due to spleen-qi deficiency. *Journal of Acupuncture and Tuina Science*.

[19] Chu H., Huang X., Li X., Chen H., Ding Y. (2011). Moxibustion for diarrhea-type irritable bowel syndrome: a clinical study. *Journal of Anhui TCM College*.

[20] Chen S. X., Wang Y. W. (2013). Thirty two cases of diarrhea-predominant irritable bowel syndrome treated by heat-sensitive moxibustion. *Zhejiang Journal of Traditional Chinese Medicine*.

[21] Ma Y.-X., Liu X., Liu C.-Z. (2013). Randomized clinical trial: the clinical effects of herb-partitioned moxibustion in patients with diarrhoea-predominant irritable bowel syndrome. *Evidence-Based Complementary and Alternative Medicine*.

[22] Higgins J. P. T., Green S. (2011). *Cochrane Handbook for Systematic Reviews of Interventions*.

[23] Luo J., Leng W. D. (2013). *Theory & Practice of Systematic Review/Meta-Analysis*.

[24] Higgins J. P. T., Thompson S. G. (2002). Quantifying heterogeneity in a meta-analysis. *Statistics in Medicine*.

[25] Cash B. D., Chey W. D. (2005). Diagnosis of irritable bowel syndrome. *Gastroenterology Clinics of North America*.

[26] Drossman D. A., Dumitrascu D. L. (2006). Rome III: new standard for functional gastrointestinal disorders. *Journal of Gastrointestinal and Liver Diseases*.

[27] Zhou S. Q., Li D. G. (2007). The epidemic survey of Fujian teenagers with irritable bowel syndrome. *Chinese Journal of Digestive Diseases*.

[28] Longstreth G. F., Thompson W. G., Chey W. D., Houghton L. A., Mearin F., Spiller R. C. (2006). Functional bowel disorders. *Gastroenterology*.

[29] Bundeff A. W., Woodis C. B. (2014). Selective serotonin reuptake inhibitors for the treatment of irritable bowel syndrome. *Annals of Pharmacotherapy*.

[30] Zakko S., Barton G., Weber E., Dunger-Baldauf C., Rühl A. (2011). Randomised clinical trial: the clinical effects of a novel neurokinin receptor antagonist, DNK333, in women with diarrhoea-predominant irritable bowel syndrome. *Alimentary Pharmacology and Therapeutics*.

[31] Ortiz-Lucas M., Tobías A., Saz P., Sebastián J. J. (2013). Effect of probiotic species on irritable bowel syndrome symptoms: a bring up to date meta-analysis. *Revista Espanola de Enfermedades Digestivas*.

[32] Rezaie A., Nikfar S., Abdollahi M. (2010). The place of antibiotics in management of irritable bowel syndrome: a systematic review and meta-analysis. *Archives of Medical Science*.

[33] Dorn S. D., Kaptchuk T. J., Park J. B. (2007). A meta-analysis of the placebo response in complementary and alternative medicine trials of irritable bowel syndrome. *Neurogastroenterology and Motility*.

[34] Qi L., Liu H.-R., Yi T. (2013). Warming moxibustion relieves chronic visceral hyperalgesia in rats: relations to spinal dynorphin and orphanin-FQ system. *Evidence-Based Complementary and Alternative Medicine*.

[35] Zhou E.-H., Wang X.-M., Ding G.-H. (2011). Suspended moxibustion relieves chronic visceral hyperalgesia and decreases hypothalamic corticotropin-releasing hormone levels. *World Journal of Gastroenterology*.

[36] Wu L. Y., Bao C. H., Ge L. B. (2011). Mild moxibustion at tianshu (ST 25) decreases expression of prokineticin-1 and prokineticin receptor-1 in colon tissue of rats with chronic visceral hyperalgesia. *Neural Regeneration Research*.

[37] Liu H. R., Hua X. G., Yang Y., Wu H. G. (2006). Clinical study on 5-HT expression in colonic mucosa and the treatment of herb-partition moxibustion in diarrhea-predominant IBS. *Liaoning Zhongyi Zazhi*.

[38] Saito Y. A., Schoenfeld P., Locke G. R. (2002). The epidemiology of irritable bowel syndrome in North America: a systematic review. *The American Journal of Gastroenterology*.

[39] Park J.-W., Lee B.-H., Lee H. (2013). Moxibustion in the management of irritable bowel syndrome: systematic review and meta-analysis. *BMC Complementary and Alternative Medicine*.

[40] Wu H. G., Zhao C., Shi Z., Chen H. P., Liu Y., Liu S. M. (2002). Clinical study on spleen-stomach-reinforcing moxibustion treatment of diarrhea-type irritable bowel syndrome. *World Journal of Acupunct-Moxibust*.

[41] Wilson A., Longstreth G. F., Knight K. (2004). Quality of life in managed care patients with irritable bowel syndrome. *Managed Care Interface*.

[42] Park J. M., Choi M.-G., Kim Y. S. (2009). Quality of life of patients with irritable bowel syndrome in Korea. *Quality of Life Research*.

